# Reprotoxicity of the Antifoulant Chlorothalonil in Ascidians: An Ecological Risk Assessment

**DOI:** 10.1371/journal.pone.0123074

**Published:** 2015-04-13

**Authors:** Alessandra Gallo, Elisabetta Tosti

**Affiliations:** Department of Biology and Evolution of Marine Organisms, Stazione Zoologica Anton Dohrn, Naples, Italy; Duke University Marine Laboratory, UNITED STATES

## Abstract

Chlorothalonil is a widely used biocide in antifouling paint formulation that replaces tin-based compounds after their definitive ban. Although chlorothalonil inputs into the marine environment have significantly increased in recent years, little is known about its effect on marine animals and in particular on their reproductive processes. In this line, the aim of the present study was to investigate the effects of chlorothalonil exposure on the gamete physiology, fertilization rate and transmissible damage to offspring in the marine invertebrate *Ciona intestinalis* (ascidians). To identify a possible mechanism of action of chlorothalonil, electrophysiological techniques were used to study the impact on oocyte membrane excitability and on the electrical events occurring at fertilization. The pre-exposure of spermatozoa and oocytes to chlorothalonil did not affect the fertilization rate but caused damage to the offspring by inducing larval malformation. The highest toxicity was observed when fertilization was performed in chlorothalonil solutions with the lowest EC_50_ value. In particular, it was observed that low chlorothalonil concentrations interfered with embryo development and led to abnormal larvae, whereas high concentrations arrested embryo formation. In mature oocytes, a decrease in the amplitudes of the sodium and fertilization currents was observed, suggesting an involvement of plasma membrane ion currents in the teratogenic mechanism of chlorothalonil action. The risk estimation confirmed that the predicted no-effect concentration (PNEC) exceeded the predicted effect concentration (PEC), thus indicating that chlorothalonil may pose a risk to aquatic species.

## Introduction

The widespread use of antifouling paints containing the biocide tributyltin (TBT) to prevent the settlement and growth of organisms on submerged structures has caused several adverse effects, such as reproductive disorders in different marine species [1]. Therefore, in the last decade, new environmentally friendly organic booster biocides have been developed as TBT alternatives.

These antifoulants are intended to be less harmful for the environment, however, their fate and toxicity in the marine environment are poorly known and, at present, there is a growing concern that they may exert the same adverse environmental effects as TBT [2, 3].

Chlorothalonil (2,4,5,6-tetrachloroisophthalonitrile) is among the most used organic booster biocides [4] and has already been employed as a fungicide in agriculture, wood protection, insecticide and acaricide [5]. Due to the wide range of applications, chlorothalonil inputs into the marine environment have significantly increased in recent years [6, 7]. In fact, elevated concentrations of this biocide have been detected in marine environments worldwide, ranging from 0.031nM (0.008 μg/l) up to 108 nM (29.78 μg/l) [7–10].

The toxic effects of chlorothalonil have been reported for marine vertebrates and invertebrates. Bioassays on embryos, larvae, juvenile and adults showed that chlorothalonil induces adult and larval mortality in fishes [11, 12], crustaceans [12–14] and mollusks [11], in addition to anomalies in the embryo and larval development of ascidians, echinoderms and mollusks [15].

In spite of the high environmental levels of chlorothalonil, limited data are available on its potentially adverse impact on marine animals, and in particular on marine invertebrates [3, 16–18]. This is alarming in view of the ecological importance of invertebrates, which are key components of all ecosystems [19].

It is well known that pollution may impair reproductive success by acting on gamete quality [20]. In particular, in marine animals characterized by external fertilization gametes are spawned into seawater and consequently exposed to water pollutants that, by altering their physiology, may seriously affect fertilization success and embryo development and in turn the reproductive fitness up to survival of the species [21, 22]. To date, no data are available on gamete impairment induced by chlorothalonil or on related alterations in fertilization success and larval development.

The ascidian *Ciona intestinalis* is a hermaphroditic broadcast spawner; consequently, its gametes are released in the sea water and are vulnerable targets for pollutants, as it is the entire fertilization process. The reproductive physiology of this ascidian is well known. In particular, the electrical properties of the oocyte plasma membrane and the ion currents involved in the fertilization process have been well described [23–25]. The mature oocyte, arrested to metaphase I (MI), is characterized by the prevalence of sodium (Na^+^) currents. At fertilization, the first electrical modification of the oocyte is the change in ion permeability due to the gating of nonspecific ion channels generating an ion current across the plasma membrane, defined as the fertilization current (FC) [26, 27]. Approximately 50 min after fertilization, the zygote undergoes the first cleavage and the following divisions occur every 35 min up to a swimming larva that hatches from the extracellular investments after 24 h post-fertilization. Since *C*. *intestinalis* has been recognized as a suitable biological model for environmental toxicological studies [28, 29], here it was chosen to investigate the potential toxicity of chlorothalonil on the gametes and fertilization by evaluating, for the first time, the effects of this biocide on the oocyte ion current activity and the electrical events of fertilization.

Finally, the predicted no-effect concentration (PNEC) was calculated and compared to the predicted effect concentration (PEC) to estimate the ecological risks associated with the occurrence of chlorothalonil in the marine environment.

## Materials and Methods

If not otherwise stated, chemicals were purchased from Sigma-Aldrich (Milan, Italy).

### Ethics statement

The research described herein was performed on the marine invertebrate *Ciona intestinalis* collected in the Gulf of Naples, Italy, (40°58'0''north latitude, 13°59'47''east longitude), a location that is not privately-owned nor protected in any way, according to the authorization of Marina Mercantile (DPR 1639/68, 09/19/1980, confirmed on 01/10/2000). This ascidian is not protected by any environmental agency in Italy and this study was carried out in strict accordance with European (Directive 2010/63) and Italian (Decreto Legislativo n. 116/1992) legislation for the care and use of animals for scientific purposes.

### Biological material

After collection, adults of *C*. *intestinalis* sp. A [30] were transported to the laboratory and maintained in aquaria with running seawater at 18°C for at least 2 days until the experiments. Animals were anesthetized in ice and mature oocytes were dissected from the oviduct and maintained in Petri dishes containing artificial seawater until further analyses (ASW: 400 mm NaCl; 50 mm MgCl_2_; 10 mm KCl; 10 mm CaCl_2_; 10 mm Hepes; pH adjusted to 8.2 with NaOH). Spermatozoa were collected with a Pasteur pipette from the sperm duct and diluted to 10^6^/ml in ASW before insemination.

### Test solutions

Stock solutions were prepared by dissolving chlorothalonil (highest available purity, Residue Analysis-Pestanal) in dimethyl sulfoxide (DMSO). Test solutions were then obtained by diluting the stock solution in ASW as follows: 1, 5, 10, 50, 100, 500, 1000, 2000, 3000 and 5000 nM. These concentrations were chosen based on the literature toxicity data [8, 15]. As DMSO was used as solvent, a negative control was prepared with an equivalent volume to a final concentration of 0.1% (i.e., the higher concentration in the test solutions), Each experiment was repeated ten times in triplicate.

### Spermiotoxicity test

Freshly collected spermatozoa were diluted in ASW at a final concentration of 10^6^/ml. Aliquots of this dilution were added to vials containing 1 ml of test solutions and incubated for 30 min at room temperature. Following exposure, spermatozoa were added to 10 ml of ASW containing approximately 200 oocytes. As a control, an untreated aliquot of the sperm dilution was used to fertilize oocytes from the same batch. The dishes were incubated in a culture chamber at 18°C; after 50 min, the fertilization rate was determined by the occurrence of the first cleavage in the control. Then, the dishes were incubated again for 24 h and the percentage of normal larvae was calculated. The fertilization rate and the percentage of normal larvae were determined on a sample of 200 oocytes.

### Oocyte toxicity test

Mature oocytes (ca 200), collected from the same animal, were transferred into Petri dishes containing 5 ml of test solution and incubated for 30 min at room temperature. Oocytes were then rinsed with ASW, transferred to Petri dishes containing 10 ml of ASW and then fertilized. Control oocytes from the same batch were fertilized. The dishes with either control or exposed oocytes were incubated in a culture chamber at 18°C. Fertilization rate and the percentage of normal larvae were determined as previously described.

### Fertilization toxicity test

To test the effects of chlorothalonil on fertilization, mature oocytes (ca 200 oocytes) and spermatozoa were added to Petri dishes containing 10 ml of chlorothalonil solutions. As a control, the two gametes were added to Petri dish containing ASW. The dishes were incubated in a culture chamber at 18°C. Fertilization rate and the percentage of normal larvae were determined as above.

### Electrophysiological recording

To evaluate the impact of chlorothalonil on oocyte ion currents, electrophysiological recordings were performed by using the whole-cell configuration of the patch-clamp technique.

The chorion and follicle cells surrounding the mature oocytes were removed manually using steel needles and naked oocytes were then transferred to a recording chamber containing ASW.

Recordings were performed using a List EPC-7 patch-clamp amplifier (HEKA Electronics, Cologne, Germany) filtered at 3 kHz and digitized with a Digidata 1322A. Ion currents were acquired and analyzed using pClamp9 software (Axon Instruments, Union City, CA, USA).

Patch pipettes were made from borosilicate glass capillaries (Warner Instruments, Hamden, CT, USA) and pulled using a Sutter P-87 (Sutter Instrument, Novato, CA, USA) with a tip of 1–2 μm in diameter showing a resistance of 3–5 megaohms when filled with an intracellular-like solution (200 mM K_2_SO_4_; 20 mM NaCl; 200 mM sucrose; 10 mM ethylene glycol tetraacetic acid (EGTA); 10 mM HEPES, pH adjusted to 7.5).

Electrophysiological recordings were performed as follows: after formation of a cell-attached configuration, a light negative pressure was applied to induce the rupture of the membrane patch allowing access to the oocyte cytoplasm. In this configuration, from a holding potential of -80 mV, voltage-dependent Na^+^ currents were elicited by applying depolarizing ramps of 10 mV to the test potential from -70 mV to +20 mV to generate the current-voltage relationship (I/V curves). These currents were maximally activated by a voltage step to -40 mV. The FC was generated by adding spermatozoa to the recording bath containing an oocyte voltage clamped at -80 mV.

To evaluate the effect of chlorothalonil on Na^+^ currents and FC, the oocytes were incubated for 30 min in test solutions before the electrophysiological recording.

### Risk estimation

To estimate the environmental risk posed by chlorothalonil, the risk quotients (RQs) were determined by comparing the PEC to the PNEC. According to other authors, it has been considered a PEC value of 1.4 μg/l as the worst-case concentration [15]. The PNEC value combines toxicity data with an assessment factor. Based on the acute toxicity data obtained from the present study, the PNEC was derived by dividing the obtained median effective concentration (EC_50_) value with an assessment factor of 1,000 as recommended by the European Commission [31]. An RQ greater than 1.0 indicates a potential environmental concern for ascidian species.

### Statistical analysis

Data were reported as the mean ± standard deviation (SD). To test for significant differences between the control group and test concentrations and among the test concentration groups, one-way analysis of variance (ANOVA) followed by Fisher’s least significant difference (LSD) test were performed. In the case of values expressed as percentages, the data were analyzed after arcsine transformation to achieve normality. The significance level was always set at α = 0.05.

The probit method was used to calculate the EC_50_, defined here as the toxicant concentration that reduced normal larvae by 50%; next, the data were normalized to the control mean percentage of normal larvae using Abbot’s formula [32].

## Results

Control experiments carried out with the selected solvent, DMSO, exhibited no observable effect on fertilization rate and embryo development.

### Spermiotoxicity test

**Fig 1:**
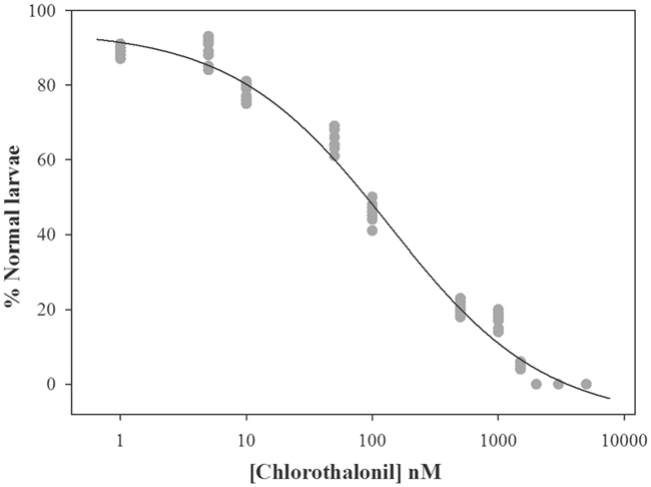
Chlorothalonil spermiotoxicity in *C*. *intestinalis*. Percentage of normal larvae after 30 min of sperm exposure to different concentrations of chlorothalonil. Solid circles represent the mean of three replicates for each experiment, and the solid line indicates the fit of the experimental data to the logistic model.

The pre-exposure of fertilizing spermatozoa to chlorothalonil had no significant effect on fertilization rate (Table 1) but significantly affected the development of normal larvae. At concentration of 10 nM chlorothalonil, a significant decrease of approximately 20% of the normal larvae was observed (Fig 1). By increasing the chlorothalonil concentration, the normal larvae percentage further decreased up to 2,000 nM, where the maximum reduction of normal larvae percentage was observed. The EC_50_ value was calculated at the concentration of 90 nM.

**Table 1:** Fertilization rate for spermiotoxicity test.

Chlorothalonil concentration (nM)	Fertilization Rate (%) ± SD
1	90.51 ± 2.2
5	89.63 ± 3.1
10	88.54 ± 2.8
50	86.32 ± 2.2
100	87.91 ± 2.9
500	88.78 ± 2.5
1000	89.10 ± 2.7
2000	90.32 ± 1.9
3000	88.95 ± 2.4
5000	89.84 ± 2.1

Mean ± standard deviation (SD) of fertilization rate calculated after sperm exposure to different concentrations of chlorothalonil.

### Oocyte toxicity test

**Fig 2:**
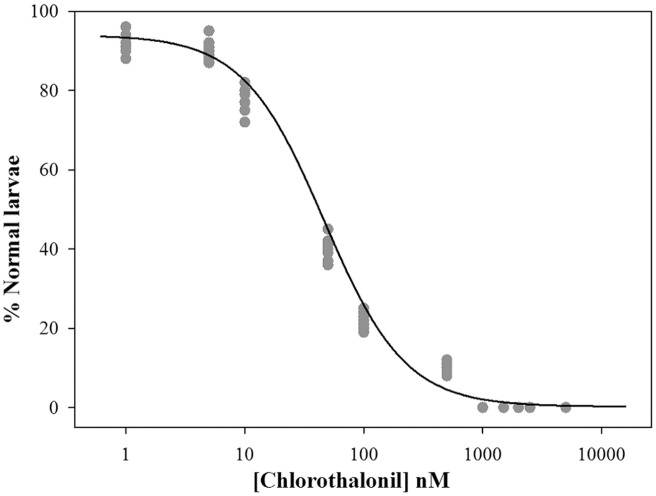
Chlorothalonil oocyte toxicity in *C*. *intestinalis*. Percentage of normal larvae after 30 min of oocyte exposure to different concentrations of chlorothalonil. Solid circles represent the mean of three replicates for each experiment, and the solid line indicates the fit of the experimental data to the logistic model.

The fertilization rate did not significantly change after oocyte exposure to chlorothalonil (Table 2); however, the percentage of normal larvae decreased in a concentration-dependent manner. The normal larvae percentage was significantly reduced at 10 nM and progressively decreased with the increase in the chlorothalonil concentration, reaching 100% abnormal larvae at 1,000 nM with an EC_50_ value of 42 nM (Fig 2).

**Table 2:** Fertilization rate for oocyte toxicity test.

Chlorothalonil concentration (nM)	Fertilization Rate (%) ± SD
1	88.35 ± 2.5
5	88.71 ± 2.0
10	87.96 ± 2.2
50	89.11 ± 2.6
100	87.88 ± 1.9
500	87.63 ± 2.1
1000	88.58 ± 2.4
2000	89.33 ± 2.5
3000	88.85 ± 2.3
5000	87.49 ± 2.0

Mean ± standard deviation (SD) of fertilization rate calculated after oocyte exposure to different concentrations of chlorothalonil.

### Fertilization toxicity test

**Fig 3:**
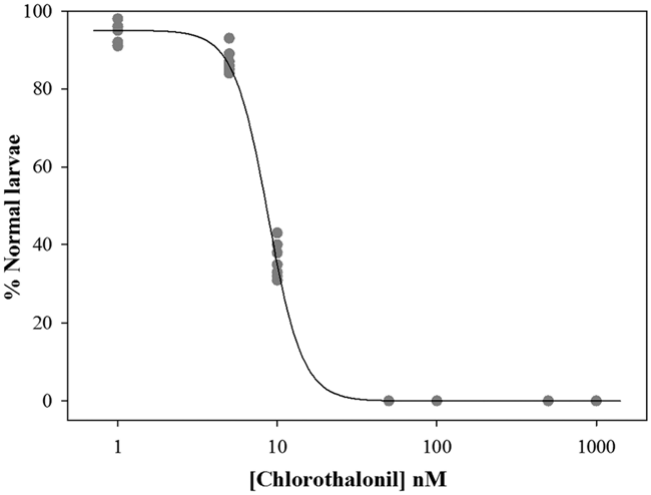
Chlorothalonil effect on *C*. *intestinalis* fertilization process. Percentage of normal larvae after fertilization in chlorothalonil solution. Solid circles represent the mean of three replicates for each experiment, and the solid line indicates the fit of the experimental data to the logistic model.

No significant effects on the fertilization rate were observed when fertilization was performed in the chlorothalonil solutions (Table 3). However, a significant decrease in the percentage of normal larvae was detected by increasing the chlorothalonil concentration from 5 nM up to 50 nM, which resulted in the absence of normal larvae with an EC_50_ value of 8.5 nM (Fig 3).

**Table 3:** Fertilization rate for fertilization toxicity test.

Chlorothalonil concentration (nM)	Fertilization Rate (%) ± SD
1	89.88 ± 1.9
5	90.05 ± 2.6
10	88.73 ± 2.1
50	89.55 ± 2.0
100	87.92 ± 2.7
500	88.42 ± 2.3
1000	90.17 ± 2.1
2000	89.11 ± 2.2
3000	89.33 ± 2.2
5000	88.92 ± 2.5

Mean ± standard deviation (SD) of fertilization rate calculated when fertilization occurs in different concentrations of chlorothalonil.

In addition, increasing chlorothalonil concentrations arrested the embryo development at earlier stages. At concentrations between 5 and 50 nM, the embryos developed into hatched larvae, however they showed abnormal curled tails and lacked one of the sensory organs.

**Fig 4:**
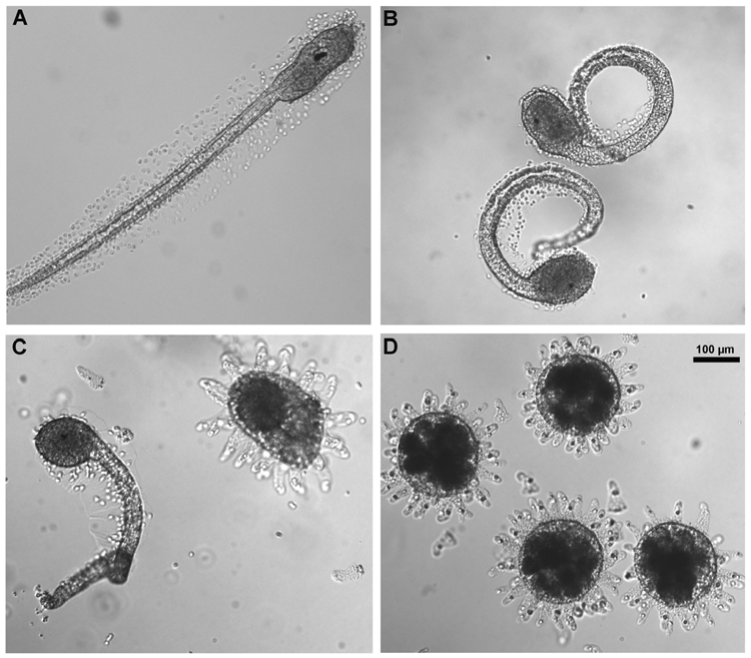
Chlorothalonil effect on *C*. *intestinalis* larvae morphology. (A) Normal larvae at 24 h post fertilization developed from oocytes fertilized in NSW. (B) Abnormal larvae developed from oocytes fertilized in chlorothalonil solution at concentrations between 5 nM and 50 nM showed curled tails and the absence of one sensory organ. (C) Abnormal larvae and embryo arrested at the blastula stage at chlorothalonil concentrations between 50 and 100 nM. (D) Embryos stopped at 8-cell stage at chlorothalonil concentrations higher than 100 nM. Scale bar is 100 μm.

At concentrations between 50 and 100 nM, it was found both abnormal larvae and embryos arrested at the blastula stage. At concentrations higher than 100 nM, chlorothalonil induced the arrest of embryo development at the 8-cell stage (Fig 4).

### Chlorothalonil effects on oocyte Na^+^ current amplitude

**Fig 5:**
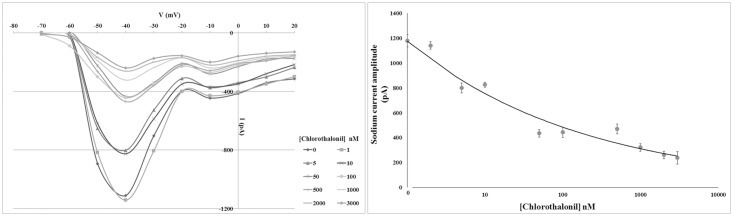
Chlorothalonil effect on oocyte Na+ current amplitude. Left panel: I/V curves constructed from current peak values recorded in oocytes clamped from the holding potential of -80 mV to the test potentials between -70 mV and +20 mV after incubation for 30 min in different chlorothalonil concentrations. Error bars indicating the SD were omitted for image clarity. Right panel: Na^+^ current amplitude recorded at the test potential of -40 mV decreased in a concentration-dependent manner. Error bars indicate the SD.

The I/V curves constructed from oocytes clamped at different voltage values and treated with the test concentrations of chlorothalonil showed that this biocide significantly decreased the amplitude of the Na^+^ current in a concentration-dependent manner starting at the concentration of 5 nM. Na^+^ current amplitude did not significantly change after incubation in 1 nM chlorothalonil while the exposure to 5 and 10 nM induced a significant reduction of Na^+^ current amplitude. At concentrations between 50 and 500 nM, the amplitude of these currents decreased again. By increasing the chlorothalonil concentration (1,000 nM), a further significant reduction in the Na^+^ current amplitude was observed up to a plateau at the 2,000 nM chlorothalonil concentration (Fig 5 and Table 4).

**Table 4:** Chlorothalonil effects on oocyte Na^+^ current amplitude.

Chlorothalonil concentration (nM)	Na^+^ current amplitude (pA) ± SD
0	1,180 ± 50
1	1,140 ± 30
5	800 ± 40
10	820 ± 20
50	430 ± 30
100	440 ± 40
500	470 ± 40
1000	320 ± 30
2000	260 ± 30
3000	240 ± 50

Mean ± standard deviation (SD) of Na^+^ current amplitude in oocytes incubated for 30 min in different concentrations of chlorothalonil.

### Chlorothalonil effects on the FC

**Fig 6:**
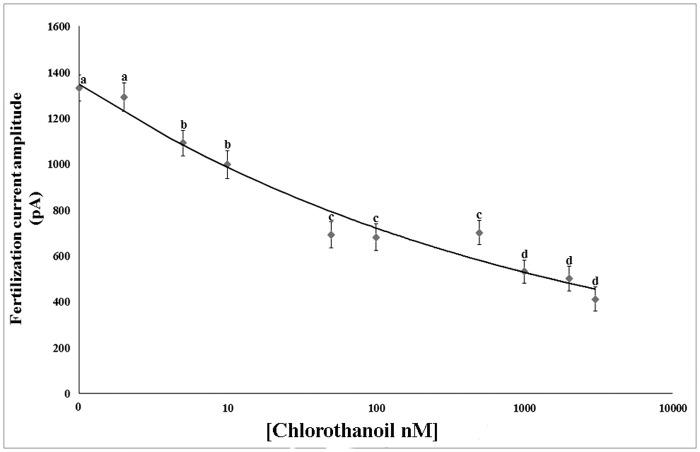
Chlorothalonil effect on fertilization current (FC) amplitude. After chlorothalonil treatment, where oocytes were clamped at -80 mV and fertilized, the FC amplitude was reduced in a concentration-dependent manner. Each experimental treatment and control was executed ten times in triplicates. ^a-b-c-d^ Different superscripts denote highly significant difference, p <0.01. Error bars indicate the SD.

In oocytes treated with chlorothalonil and then fertilized under voltage clamp conditions, the frequency of the FC showed no significant differences between the control and the test concentration groups. By contrast, the FC amplitude significantly decreased in a concentration-dependent manner (Fig 6). FC amplitude did not significantly change in oocytes treated with 1 nM chlorothalonil. In oocytes treated with 5 nM and 10 nM chlorothalonil, the FC amplitude significantly decreased. By increasing the chlorothalonil concentration (50 nM), the FC amplitude decreased again and no differences were observed at concentrations of 100 nM and 500 nM. However, by further increasing chlorothalonil concentrations (1,000 and 2,000 nM), we obtained a significant reduction in the FC. The minimum amplitude of the FC was recorded at 3,000 nM chlorothalonil (Table 5).

**Table 5:** Chlorothalonil effects on the fertilization current (FC).

Chlorothalonil concentration (nM)	FC amplitude(pA) ± SD
0	1,330 ± 58
1	1,290 ± 62
5	1,090 ± 55
10	997 ± 61
50	690 ± 57
100	680 ± 59
500	700 ± 52
1000	530 ± 50
2000	500 ± 54
3000	410 ± 52

Mean ± standard deviation (SD) of fertilization current (FC) amplitude in oocytes incubated for 30 min in different concentrations of chlorothalonil and then fertilized.

## Discussion

In the present study, the toxicity of the biocide chlorothalonil to *C*. *intestinalis* gametes and fertilization was investigated. The obtained results showed that this compound significantly reduces both the Na^+^ and fertilization currents and affects larval development. Following the worldwide ban on the use of TBT as an antifouling agent, organic booster biocides, which have already been used in other applications, have been introduced as alternative active ingredients in paint formulations. Therefore, it was expected that these compounds should not be toxic to marine organisms while maintaining the same efficiency against fouling; however, concerns have been expressed that these biocides could also cause serious environmental damages [33]. Although there is substantial information to support their ecotoxicity, additional data are still required to evaluate the risk associated with the occurrence of organic booster biocides in the marine environment due to their widespread and increasing use.

Although the adverse effects of new antifouling biocides have received increased attention, few studies have investigated the toxicity of these compounds on the reproductive processes of marine organisms. The sperm toxicity of several alternative antifoulants, such as irgarol, diuron, copper pyrithione, dichlofluanid, tolylfluanid and sea-nine, has been investigated only in oysters and sea urchins. These studies revealed that all the tested biocides acted as spermiotoxic agents [34–38]. On the other hand, information on the toxicity of antifouling biocides on marine organism oocytes are available only for zinc pyrithione [28]. Although chlorothalonil is at present one of the most employed antifouling compounds, data concerning its toxicity on marine invertebrates are still scarce.

Here, it was demonstrated that *C*. *intestinalis* spermatozoa exposed to chlorothalonil did not show an impaired fertilization capability but gave rise to a significant decrease in the normal larvae percentage with an EC_50_ value of 90 nM, indicating that treated spermatozoa retain fertilization abilities but produce offspring that develop abnormally. Moreover, the pre-exposure of oocytes to chlorothalonil induced transmissible damage and larval malformations with an EC_50_ value of 42 nM, without affecting the fertilization rate. Previously, similar transmissible damage of *C*. *intestinalis* oocytes exposed to zinc pyrithione with a higher EC_50_ value was found [28]. By contrast, a recent study demonstrated that diuron, another biocide of new generation, did not exert adverse effects on *C*. *intestinalis* spermatozoa and oocytes [22]. In the present study, chlorothalonil appears to be the most toxic antifouling compound for gametes of the ascidian *C*. *intestinalis*, as also previously reported for the embryo and larvae of bivalves, sea urchins and ascidians [15].

The highest toxicity was observed when fertilization was performed in chlorothalonil solutions with the lowest EC_50_ value. Furthermore, increased chlorothalonil concentrations arrested *C*. *intestinalis* embryo development at earlier stages; in particular, it was observed that low chlorothalonil concentrations interfered with embryo development and led to larval malformation, whereas high concentrations completely arrested the larval formation. Previous studies have only assessed chlorothalonil effects on the embryo development starting from fertilized oocyte in various species. The EC_50_ values calculated here for *C*. *intestinalis* were within the range of values previously registered for other marine organisms. The EC_50_ value for oyster embryogenesis is 13.8 nM [5] and values of 33 and 25 nM have been recorded for blue mussel and sea urchin embryogenesis, respectively [15]. The EC_50_ value previously reported for *C*. *intestinalis* embryogenesis (123 nM) was higher than that calculated in the present study, suggesting that the fertilization process is more sensitive to the tested biocide than embryogenesis. In fact, even though fertilization rate was not affected by chlorothalonil, it is feasible that the basic mechanisms accompanying fertilization process are influenced, since it was observed a negative impact on the FC.

To date, few data are available on the mechanism of action of chlorothalonil, it has been hypothesized that a possible toxic action is due to glycolysis inhibition or glutathione depletion [39].

It has been reported that some antifouling compounds affect the functioning of voltage- and sperm- dependent ion currents of the oocyte plasma membrane. Previously, the critical role played by Na^+^ currents in the physiology of mature oocytes and the following fertilization in *C*. *intestinalis* was demonstrated [23] and a reduction of oocyte Na^+^ currents has been provided for the antifoulants TBT and diuron in the ascidians *Phallusia mammillata* and *C*. *intestinalis* [22, 40]. However, a similar impact has not been previously documented for chlorothalonil. In this study, for the first time, it was demonstrated that this biocide induces a reduction in the Na^+^ current amplitude in a concentration-dependent manner along with a decrease in the FC amplitude. FC has been recorded in many different animal species from marine invertebrates to humans; however, its functional role has not yet been clarified [41–44]. Some studies reported an involvement of the FC in exerting a long-term effect of normal embryo development in *C*. *intestinalis*. In fact, it has been shown that the inhibition of FC impairs embryo development generating abnormal larvae characterized by sensory organ pigmentation absence and malformations of the tail, which is a key feature for larval metamorphosis in ascidians [22, 23, 45].

These larval malformations resemble those observed in the present study, thus corroborating the idea that the inhibition of sperm-activated channels may exert a long-term effect on embryo development being at the base of the teratogenic mechanism of chlorothalonil.

On the basis of the results obtained, the PNEC was tentatively estimated and compared to the PEC to calculate the RQ. The RQ values were above the threshold level of 1.0 for the three performed toxicity tests suggesting a high level of risk to the ascidian *C*. *intestinalis* associated with the occurrence of chlorothalonil in the marine environment. The reproductive success of a large number of marine species is known to be harmed by biocides and in this study it has been shown that chlorothalonil exposure induces a series of reproductive disorders that may in turn alter specie fitness and survival with serious consequences for marine ecosystems.

## Conclusions

Chlorothalonil is commonly considered less dangerous than TBT because of its more rapid environmental degradation, which results in low concentrations in both sediments and seawater. In the present study, it has been shown that low concentrations of chlorothalonil can cause deleterious effects on ascidian gametes and fertilization and in turn impair embryo development. Consequently, its eligibility as an alternative antifouling biocide is questionable.

To date, the majority of bioassays using marine invertebrates have been performed with either embryos and larvae or adults, whereas the sensitivity of spermatozoa and oocytes to pollutants, and in particular to antifouling biocides, remains largely unknown. This study provides the first evidence of transmissible damage to marine invertebrate offspring induced by chlorothalonil, thus suggesting that further research is needed to evaluate its harmful effects on the gametes of other marine organisms. Although ascidians are subjected to antifouling strategies by being fouling organisms themselves, they nevertheless represent species that should be protected because they are cosmopolitan ecological components of the marine ecosystem. The fact that the gametes and fertilization process of *C*. *intestinalis* are very sensitive to environmental pollution confirms that this ascidian is a suitable models to evaluate the toxicity of marine contaminants and for seawater quality assessments.
